# Case report of thrombotic thrombocytopenic purpura during pregnancy with a review of the relevant research

**DOI:** 10.1097/MD.0000000000038112

**Published:** 2024-05-17

**Authors:** Jia Xu, Li-na Tan, Ling-xia Li, Gu-Yuan Qiao

**Affiliations:** a Department of Obstetrics and Gynecology, Xijing Hospital, Fourth Military Medical University, Xi’an, China; b Department of Obstetrics and Gynecology, Xi’an People’s Hospital, Xi’an, China.

**Keywords:** ADAMT13, plasma exchange, pregnancy, thrombotic thrombocytopenic purpura

## Abstract

**Rationale:**

Thrombotic thrombocytopenic purpura (TTP) is a syndrome characterized by widespread blood vessel clotting and bleeding. It can affect individuals of any age but is more commonly observed in females, particularly during pregnancy. Pregnancy combined with TTP is a critical and rapidly progressing condition that is often misdiagnosed as an obstetric disorder like severe preeclampsia or HELLP syndrome. To deepen the understanding of TTP during pregnancy with the help of a clinical case.

**Patient concerns:**

A 20-year-old patient, is pregnancy 1 birth 0, 32 weeks dated by her last menstrual period, presented chest tightness, and shortness of breath after physical activity for 3 days.

**Diagnoses:**

TTP.

**Interventions:**

At present, there are no preventive measures. Timely diagnosis and treatment are useful. Plasma exchange and treat to the patient hinder autoantibodies, such as gamma globulin, methylprednisolone, rituximab, and cyclosporine were effective.

**Outcomes:**

The patient exhibited stable vital signs, normal examination results, and experienced no complications. We continued to monitor her progress after she was discharged.

**Lessons subsections:**

The acute onset of TTP is often associated with pregnancy, as it is a triggering factor. Timely identification, accurate diagnosis, and a comprehensive treatment approach involving plasma exchange, immunosuppressants, and the termination of pregnancy can lead to remission and a favorable outlook for the majority of patients.

## 1. Introduction

Thrombotic thrombocytopenic purpura (TTP) is a syndrome characterized by widespread blood vessel clotting and bleeding. Its clinical features include a combination of 3 main symptoms: thrombocytopenic purpura (a decrease in platelet count leading to bleeding under the skin), microangiopathic hemolytic anemia (red blood cell destruction due to abnormal blood vessel function), and neuropsychiatric symptoms. In some cases, a pentad of symptoms may be present, which includes renal damage and fever in addition to the triad. If left untreated, TTP has a mortality rate of 90%.^[1,2]^ It can affect individuals of any age but is more commonly observed in females, particularly during pregnancy. Pregnancy combined with TTP is a critical and rapidly progressing condition that is often misdiagnosed as an obstetric disorder like severe preeclampsia or HELLP syndrome.^[3]^ It was also reported that coexistence of renal and neurological impairments, infection, and renal damage with type IV or thrombotic microangiopathy might denote a poor prognosis of TTP patients associated with systemic lupus erythematosus.^[4]^ However, prompt and accurate diagnosis along with comprehensive management involving multiple medical disciplines can lower the mortality rate. It had been suggested to test common viral infections associated with immune thrombocytopenia (ITP) and above reported (cytomegalovirus, Epstein Barr virus, parvovirus, rubella, and measles) in newly diagnosed ITP, and in the pandemic situation, for epidemiological studies addressed to clarify a possible correlation but also for a prompt diagnosis of Covid-19 (in particular for mildly symptomatic subjects) it had been suggested to test new coronavirus in children with ITP and fever and/or respiratory symptoms (in progress or recent medical history).^[5]^ In June 2019, a patient with pregnancy and TTP was admitted to our department. Early and accurate diagnosis, positive plasma exchange, and the use of immunosuppressant medication ensured patient survival.

## 2. Case Report

### 2.1. General information

The 20-year-old patient was in good health and had a pregnancy and childbirth history of 0-0-0-0. She experienced regular menstruation lasting for 5 days, with a menstrual cycle ranging from 26 to 30 days. The most recent menstruation took place on October 26, 2018.

### 2.2. Medical history

On June 7, 2019, the patient was hospitalized with the main complaints of experiencing menopausal symptoms for 32 weeks, chest tightness, and shortness of breath after physical activity for 3 days, which worsened for 1 day. Throughout her early pregnancy, she had no history of exposure to colds, poisons, drugs, or radiation. The patient did not have any previous episodes of abdominal pain or vaginal bleeding, and she did not undergo regular prenatal tests during pregnancy such as Down screening, noninvasive DNA test, fetal system examination, fetal heart ultrasound, sugar tolerance test, or thyroid function evaluation. During her pregnancy, she occasionally experienced gum bleeding, had urine that appeared pale-yellow colored in late May 2019 without an identifiable cause, normal urine volume, and no increased foam. She also had temporary blurred vision, which was not considered significant. Three days prior to her admission, she experienced shortness of breath after physical activity, which was relieved by rest. Additionally, she had nosebleeds that lasted approximately 1 hour each time but could be stopped by applying pressure. She did not experience dizziness, headaches, fever, or any disturbance of consciousness. She had previously been admitted to a local hospital for a routine blood test, which showed a hemoglobin level of 70 g/L and platelet count of 10 × 10^9^/L. Prior to that, after walking for 1 minute on a flat road, she experienced shortness of breath, chest tightness, blurred vision, and a yellowish discoloration of her face. At that time, she underwent a routine blood test at the local hospital, which revealed a hemoglobin level of 68 g/L and platelet count of 4 × 10^9^/L. On June 7, 2019, the patient: G1P0 and LOA at 32 weeks of pregnancy, with severe anemia and thrombocytopenia, was admitted to our emergency department for further treatment.

### 2.3. Signs

Upon admission, the patient underwent a physical examination, revealing the following results: body temperature of 37.2 °C, pulse rate of 84 beats/min, respiration rate of 22 breaths per minute, blood pressure reading of 123/76 mm Hg, and a BMI of 31.25. The patient presented with anasarca and icteric sclera. The examination of the heart and lungs did not reveal any abnormalities. The patient experienced abdominal distension without any pain upon pressure or rebound. The fundal height measured 29 cm, the abdominal circumference was 105 cm, and the fetus was positioned in the LOA orientation. The fetal heart rate was recorded at 150 beats/min. Additionally, a urinary catheter was inserted, and the urine appeared a pale-yellow color.

### 2.4. Results of tests

#### 2.4.1. Test results

The patient was admitted to the hospital with a routine blood test indicating severe anemia (hemoglobin 54 g/L, red blood cell count 1.74 × 10^12^/L, platelet reduction to 7 × 10^9^/L, 3.4% of red blood cell fragmentation and 9.23% of reticulocyte percentage). The results of a routine urine test showed that the erythrocyte quantitative test value was 155.50/UL, pathological tubular quantitative test value was 10.43/UL, protein qualitative test value was 3+, microscopic red blood cell count was 2+/HP, sugar qualitative test value was 3+, and erythrocyte qualitative test value was 3+. Hemolysis was not excluded. According to the results of the blood coagulation function test, the D-dimer level of the patient was 6.75 mg/L. Liver function test indicated that the value of lactic dehydrogenase (LDH) was elevated to 3640 IU/L and renal creatinine was 111 µmol/L. Results of the cardiac injury biomarker test suggested B-type natriuretic peptide (BNP) of 1737 pg/mL and Troponin I of 0.228 ng/mL. Coombs test was negative. Additional testing revealed that the titer of autoantibodies antinuclear antibody was positive (1:320), and the titer of antinuclear antibody was positive (1:100), ruling out autoimmune disorders (Table 1).

**Table 1:** Partial test results upon admission.

Items	Results		
Blood routine test	Red blood cell count: 1.74 × 10^12^/L	Hemoglobin: 54 g/L	Platelet count: 7 × 10^9^/L
	Reticulocyte percentage: 9.23%; cell fragments: 3.4%		
Blood coagulation function test	D-dimer: 6.75 mg/L		
Liver and kidney functions	LDH: 3640 IU/L	Creatinine: 111 µmol/L	
Cardiac damage	BNP: 1737 pg/mL	Troponin: 0.228 ng/mL	CK-MB: 2.5 ng/mL
Coombs test	Negative		
Urine routine test	Urine erythrocyte quantitative test: 155.50/UL; urine pathological tubular quantitative test of 10.43/UL; urine protein qualitative test: 3+; microscopic red blood cell count: 2+/HP; urine sugar qualitative test: 3+; urine erythrocyte qualitative test: 3+		
ADAMTS113 activity	Decreased to 8.9%		
Autoantibodies	Positive antinuclear antibody (1:320) and positive antinuclear antibody (1:100)		

Urine red blood cells were observed in 10 fields with a high-power microscope to calculate the average minimum to maximum number of each field, 3+: 10 cells per high-power field.

Urine sugar refers to the sugar content in the urine; +++: urine sugar content 1% to 2%.

The qualitative pair of urine protein was roughly estimated based on the concentration of urine protein, 3+: 0.2–0.5 g/100 mL.

BNP = brain natriuretic peptide, LDH = lactic dehydrogenase.

#### 2.4.2. Laboratory tests

Genetic testing showed that ADAMTS113 activity was reduced to 8.9%.

#### 2.4.3. Fetal heart monitoring

Baseline: 140 beats/min, fetal heart rate fluctuation < 5 bpm, occasional fetal heart decelerations, nonreactive NST.

#### 2.4.4. Imaging examination

After admission, necessary examinations were completed such as the ultrasound, which suggested stillbirth. The fetus had the following measurements: biparietal diameter of 7.5 cm, an abdominal diameter of 7.5 × 7.4 cm, and a femoral length of 4.2 cm. No fetal heartbeat was detected, and the placenta was situated on the posterior wall of the uterus, measuring 2.3 cm in thickness. The amniotic fluid index (AFI) was recorded as 12.5.

### 2.5. Diagnosis and treatment

**Figure 1.**
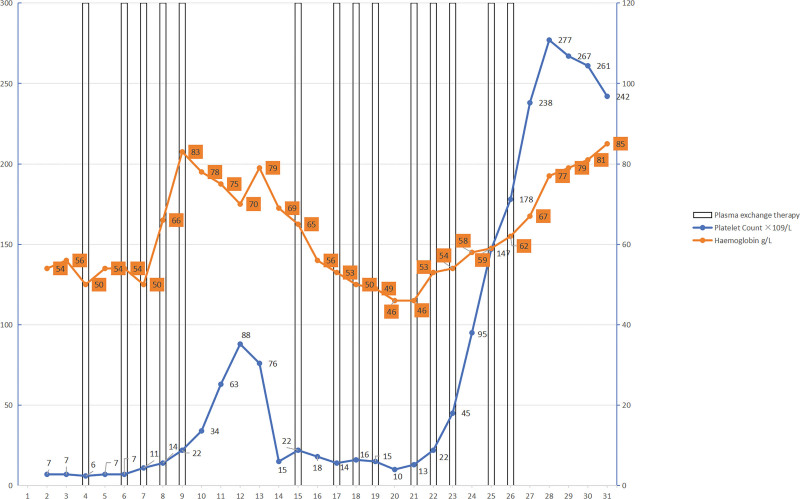
Time course of platelet count and hemoglobin concentration during treatment. The vertical lines represent plasma exchange, the blue line represents the platelet count (left *y* axis), and the red line represents the hemoglobin concentration (right *y* axis).

The patient presented with a combination of symptoms such as thrombocytopenia, hemolytic anemia, and neurological symptoms such as yellow vision in the right eye and blurred vision, which strongly suggested the presence of TTP. Following a comprehensive consultation involving specialists from the hematology, nephrology, and ophthalmology departments, the patient received plasma replacement therapy and gamma globulin for 3 days after admission. Subsequently, there was a significant improvement in all relevant indicators compared to previous measurements. There were no indications of cervical dilation or effacement. The condition of the patient was unstable, and discussions were held with the family for immediate surgery. Based on their decision, successful surgery involving both cesarean and bilateral uterine artery ligation was performed on June 10, 2019, resulting in stillbirth. However, after discontinuing plasma exchange, the patient experienced a recurrence of blurred vision and a progressive decline in both hemoglobin and platelet levels. To address these issues, the patient underwent plasma exchange and received symptomatic treatment, including gamma globulin at a dosage of 10 g/day, methylprednisolone at 80 mg/day, rituximab (a monoclonal antibody anti-CD20) at a dosage of 600 mg, and cyclosporine. These interventions led to a stable improvement in the patient’s condition. A visual representation of the changes in platelet count and blood test results can be seen in Figure 1.

### 2.6. Pregnancy outcome and follow-up

Following the administration of 14 rounds of plasma exchange, along with gamma globulin, methylprednisolone, rituximab, and cyclosporine to hinder autoantibodies, the patient was in a stable condition, and the dosage of methylprednisolone was modified to 40 mg per day. The condition of the patient was regularly monitored and evaluated.

### 2.7. Pathological examination

**Figure 2.**
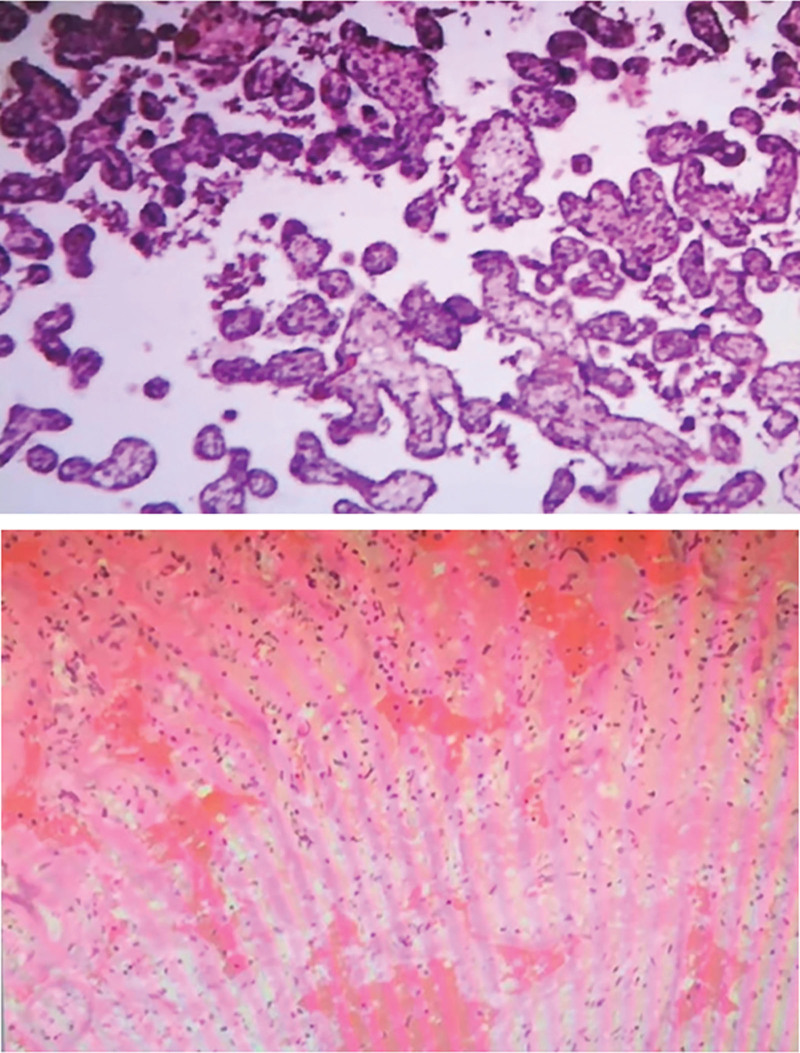
Placental umbilical cord histopathology (HE ×40).

The dimensions of the placental tissue were 15 × 12 × 3 cm, while the umbilical cord measured 50 cm in length and had a diameter of 1.7 cm. The surface of the umbilical cord appeared gray-red with visible villi and showed signs of local congestion. Upon examination using light microscopy, trophoblast cells with placental villi were observed along with localized fibrin-like necrosis. There was also evidence of necrosis in the fetal membrane and thrombosis in the umbilical cord. Refer to Figure 2 for visual representation.

## 3. Discussion

TTP results from either a congenital or acquired absence/decrease of the von Willebrand factor (vWF)-cleaving protease ADAMTS13 (a disintegrin and metalloproteinase with a thrombospondin type 1 motif member 13). Low levels of ADAMTS13 activity result in microthrombi formation, which leads to end-organ ischemia and damage. The central nervous system and kidneys are the 2 most common organ systems affected by TTP.^[6]^

TTP can be inherited or acquired, and hereditary TTP is brought on by mutations in the gene for the vWF cleavage protease (also known as *ADAMTS13*, or A Disintegrin and Metalloprotease with Thrombospondin Type 1 Motif, 13th member).^[1,2]^ Thus far, more than 150 distinct mutations have been reported, of which approximately 60% of reported mutations are missense, 20% are minor fragment deletions and insertions, and a very low rate of nonsense mutations and splicing site mutations.^[1,7]^ Depending on whether the primary disease is present in the patient, acquired TTP is further divided into idiopathic and secondary TTP, and is associated with a large release of vWF and relatively insufficient vWF cleavage protease activity (*ADAMTS13*).^[1]^ Acquired TTP accounts for approximately 95% of all TTP cases.^[8]^

In patients with hereditary TTP, there is a 100% risk of recurrence during pregnancy. Regular plasma transfusions can help prevent acute TTP attacks and fetal growth retardation in such cases.^[7]^ The risk of recurrence in patients with acquired TTP and severe ADAMTS13 deficiency during pregnancy is uncertain and may range from 0% to 50%.^[1,7]^

A study has shown that women experiencing their first episode of TTP during pregnancy have worse perinatal outcomes compared to those with recurrent TTP.^[4]^ In this case, the patient was previously healthy, had not undergone regular pregnancy examinations, experienced retardation in fetal development, and had the first onset of TTP at 32 weeks of gestation. The failure to diagnose and treat the condition early was one of the primary factors contributing to the poor perinatal outcome. It also led to the consideration of refractory TTP in the subsequent treatment. Pregnancy involves a complex set of immune mechanisms that can contribute to the development of TTP, whether it is hereditary or acquired.^[3,8]^ During a normal pregnancy, the levels of vWF and ADAMTS13, which are important components of the blood clotting process, are balanced. vWF plays a crucial role in human hemostasis by facilitating the adhesion of platelets to the damaged endothelium of blood vessels during the initial clotting phase. ADAMTS13, on the other hand, breaks down vWF multimers of large molecular weight into dimers or multimers with smaller molecular weight. In the last 3 months of a normal pregnancy, vWF levels increase to about 1.5 to 3.0 times higher than those in nonpregnant individuals,^[1]^ while ADAMTS13 levels gradually decrease to around 25% to 30% of their original level by late pregnancy.^[8]^

When the equilibrium is upset, increased vWF levels and decreased ADAMTS13 activity cause significant platelet and vascular hemophilia factor aggregation formation that can obstruct small blood arteries and cause end-organ ischemia. A severe deficiency of ADAMTS13 (<10%) is a specific and diagnostic characteristic of TTP. Research has demonstrated that low levels of ADAMTS13 are associated with unfavorable outcomes in pregnancy.^[9]^ Patients with autoimmune diseases (SLE, APS), pregnancy, or HELLP syndrome may experience a mild to moderate decrease in ADAMTS13 activity (12–50%).^[9]^ The clinical features of TTP, which may include fever, nausea, vomiting, abdominal discomfort, malaise, and flu-like symptoms, are rare, abrupt, and nonspecific.^[2]^ The majority of patients experience hemolytic anemia and have a platelet count below 50 × 10^9^/L.^[10]^ Blood samples may not always show fragmented red blood cells in the early stages of TTP. The thrombosis primarily affects the liver, kidneys, brain, and other organs, leading to impaired organ function, and some patients may also develop cerebral infarction.^[3]^ Transparent blood clots, which can be seen in biopsies and small blood vessels throughout the body according to autopsy findings, suggest that vascular biopsies can help with the diagnosis of TTP. Gum bleeding is a commonly observed initial symptom, and therefore, examining the blood vessels in the gum area can help identify related lesions.^[11]^ However, due to the invasive nature of this method, it is not widely utilized in clinical settings and does not provide significant assistance in rapid diagnosis. In this case, the patient did not have a fever upon admission, had a platelet count of only 1.74 × 10^9^/L, and experienced blurred vision as the sole neurological manifestation. Although fragmented red blood cells were not initially detected in the blood sample, they were observed upon reexamination as the disease progressed. In order to prevent needless blood or platelet transfusions that could increase the risk of arterial thrombosis and exacerbate the condition, it is crucial to consider the possibility of TTP into account as a differential diagnosis in pregnant people with thrombocytopenic disorders.^[1]^ At times, the symptoms of TTP can be mistaken for those of conditions such as preeclampsia, HELLP syndrome, or acute fatty liver in pregnancy, further complicating the diagnosis.^[7]^ Therefore, it is crucial to strive for prompt and accurate diagnosis. Patients diagnosed with HELLP syndrome typically exhibit consistently high blood pressure and have a prior history of preeclampsia. However, after the termination of pregnancy, the condition of patients with HELLP syndrome generally improves. In this particular case, the patient did not have a history of elevated blood pressure, and the platelet count did not show significant improvement after pregnancy termination. Therefore, it is unlikely that the patient had HELLP syndrome.

Acute fatty liver during pregnancy is characterized by abnormal liver function, primarily evidenced by increased levels of serum transaminases. Additionally, positive results are often observed in the 4 coagulation tests. Patients with this condition commonly experience gastrointestinal symptoms and less frequently present with anemia, particularly hemolytic anemia. In the given case, the patient had elevated transaminase levels, but normal coagulation, severe anemia, and no symptoms such as nausea, vomiting, or loss of appetite. Thus, the diagnosis of acute fatty liver can be essentially ruled out.

Hemolytic uremic syndrome (HUS) shares a similar mechanism and treatment with TTP. However, HUS generally leads to more severe renal impairment than TTP and often requires dialysis treatment. In the present case, the patient only exhibited mild impairment of renal function, suggesting that HUS is not the correct diagnosis. To arrive at a differential diagnosis, the medical history, physical examination, and examination results of the patient were carefully considered, as outlined in Table 2. Elevated levels of troponin are the sole indication of cardiac damage in TTP. Cardiac complications, such as myocardial infarction or acute conduction issues affecting the sinoatrial and atrioventricular nodes, are a significant cause of death in patients with TTP. Cardiac troponin level of 0.25 µg/L is a strong independent predictor of death, with a dominance ratio (OR) of 2.87 and a significance level (*P*) of .024.^[7]^ In this particular case, the patient had a troponin level of 0.228 ng/mL and a BNP level of 1737 pg/mL, which indicated some cardiac damage and the necessity for prompt treatment.

**Table 2:** Clinical manifestations and laboratory analysis of thrombotic microangiopathy.^[5]^

Differentiation points	TTP	Hemolytic uremic syndrome	HELLP syndrome	Acute fatty liver of pregnancy	Obstetric antiphospholipid syndrome
Onset time	Any time	Postpartum	>20 weeks	27–40 weeks	Any time
Fever	−/+	−/+	–	−/+	−/+
Abdominal symptoms	−/++	−/++	−/++	−/++	−/+
Neurological symptoms	+/+++	−/+	−/+	−/++	−/++
Decreased kidney function	−/++	+/+++	−/+	−/+	−/+
Hypertension	−/+	−/++	++/+++	−/+	+/++
Microvascular hemolytic anemia	+++	++/+++	+/+++	−/+	+
Platelets	+++	++	++	−/+	++
Lactate dehydrogenase	+++	++/+++	++/+++	+/++	+
Troponin	+/++				
Liver enzymes	−/+	−/+	++/+++	+++	+
Blood glucose	–	–	–	−/+++	–
Urine protein		++	++	−/+	−/++
Coagulation	–	–	−/+	++	−/++
ADAMTS13 activity	Severe functional deficiency (<10%)	Normal or mildly decreased	Normal or mildly decreased	Normal	Normal or mildly decreased

TTP = thrombotic thrombocytopenic purpura.

−: the symptom or sign is not present; +: the symptom or sign may be present; ++: the symptom or sign is present; +++: the main manifestations are the symptoms and signs.

ADAMTS13 activity < 10% confirms the diagnosis of TTP, and in this case, the value was 8.9%. The diagnosis of the patient was clear, and positive plasma exchange and immunosuppressive therapy resulted in improvement in anemia and thrombocytopenia, along with an increase in troponin levels and a decrease in BNP levels. However, the condition recurred upon discontinuation of plasma exchange and was stabilized by resuming plasma exchange and administering the antitumor drug rituximab. Pregnancy combined with TTP leads to a high risk of maternal and fetal mortality. In untreated cases, the mortality rate for TTP is around 90%. However, recent advancements in understanding the pathogenesis of TTP and the use of plasma exchange therapy have significantly reduced the mortality rate from 10% to 20%.^[2,9]^ A complete response to treatment is defined by a platelet count above 150 × 10^9^/L for 2 consecutive days, together with normal or normalizing LDH and clinical recovery.^[12]^ New drugs including N-acetylcysteine, bortezomib, recombinant ADAMTS13, and caplacizumab show promise in the management of TTP.^[13–18]^ Caplacizumab, an anti-vWF humanized, bivalent variable-domain-only immunoglobulin fragment, which inhibits interaction between vWF multimers and platelets. Results of clinical trials in caplacizumab showed that among patients with TTP, treatment with caplacizumab was associated with faster normalization of the platelet count; a lower incidence of a composite of TTP-related death, recurrence of TTP, or a thromboembolic event during the treatment period; and a lower rate of recurrence of TTP during the trial than placebo.^[18]^

Currently, plasma exchange is the sole effective treatment for pregnant patients with TTP. The recommended dosage is a minimum of 2000 mL per dose (40–60 mL/kg) administered 1 to 2 times per day. In this case, the patient received 2000 mL per dose, twice a day. Upon admission, the patient had severe anemia with a blood platelet count of 7 × 10^9^/L and an abnormal fetal heart rate baseline. Emergency termination of the pregnancy poses risks to both the mother and the child’s life. Examination revealed a stillborn fetus, and after plasma exchange treatment, the patient’s overall condition improved, leading to the removal of the fetus through cesarean section. Postoperative pathological examination indicated localized fibrinoid necrosis of the placenta, localized necrosis of the fetal membranes, umbilical cord thrombosis, and findings consistent with placental infarction, fetal growth retardation, and stillbirth caused by TTP. Differentiating between TTP and HELLP syndrome can be challenging clinically, as the ADAMTS13 assay does not aid in early diagnosis. Therefore, in suspected cases of TTP, diagnosis relies on careful comparison and evaluation of clinical parameters such as time of onset, symptoms, and laboratory tests.

Treatment for presumed TTP is often necessary when ADAMTS13 levels have not been measured,^[5,7]^ followed by monitoring the platelet count and levels of lactate dehydrogenase and troponin. In the current study, the patient was given a daily oral dose of 40 mg of methylprednisolone. To prevent the potential development of hereditary TTP,^[4]^ which has been associated with the use of oral contraceptives and hormone replacement therapy, an estrogen-free contraceptive was provided during follow-up whenever contraception was needed. It was recommended to closely monitor the patient’s obstetric health in subsequent pregnancies. This involved regular assessments of blood count, lactate dehydrogenase levels, binding bead protein levels, fetal growth, and placental function using fetal ultrasound.

ADAMTS13 activity should be evaluated every 3 months to determine if the therapy should be initiated before a TTP episode occurs. If there is a decline in ADAMTS13 activity, more frequent monitoring is required. Plasma exchange and immunosuppressive medication should be started if ADAMTS13 activity drops below 10%. Upon presence of significant symptoms, the frequency of plasma exchange should be increased to daily, and an early clinical diagnosis of TTP must be considered.

## 4. Conclusion

**Figure 3.**
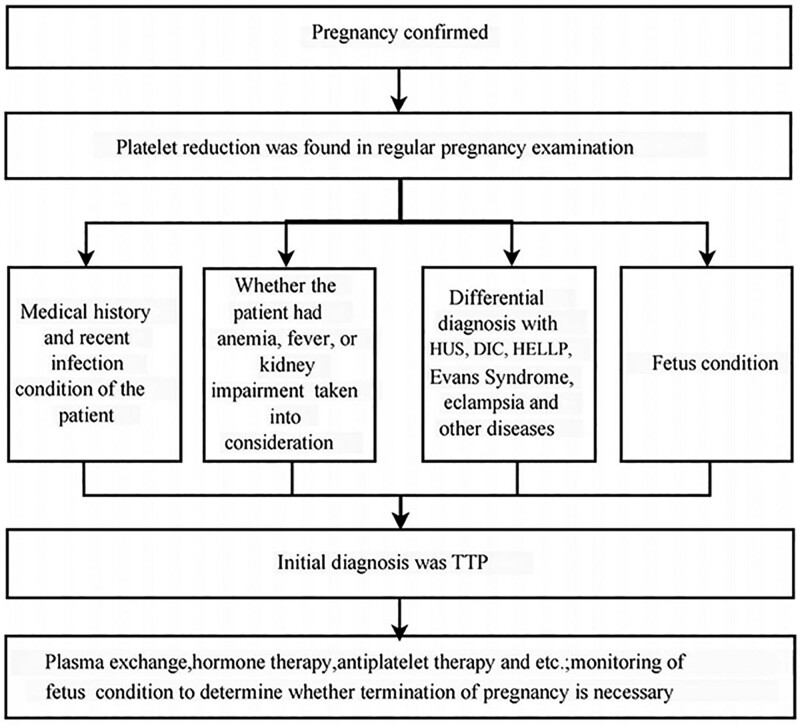
Flow chart of reported case.

Based on the current case report (flow chart shown in Fig. 3), it is recommended to perform regular examinations after pregnancy. For patients with thrombocytopenia, disease assessment should be carried out to determine whether there are induced causes, such as viral infection, anemia, neurological symptoms, fever, kidney impairment, etc, and for diagnosis and differential diagnosis should be made while considering laboratory test results at the same time. ADAMTS13 activity should be closely monitored in patients with a history of acute TTP, and plasma infusion should be given if ADAMTS13 activity is <10% and antibody (inhibitor) is negative. Immunosuppressive drugs should be given to those with positive inhibitors, if necessary.

## Acknowledgments

We would like to acknowledge the hard and dedicated work of all the staff that implemented the intervention and evaluation components of the study.

## Author contributions

**Conceptualization:** Jia Xu, Gu-Yuan Qiao

**Writing – original draft:** Jia Xu, Gu-Yuan Qiao.

**Data curation:** Li-na Tan, Gu-Yuan Qiao.

**Formal analysis:** Li-na Tan, Ling-xia Li.

**Writing – review & editing:** Ling-xia Li, Gu-Yuan Qiao.
